# Considerations on the efficacy and toxicity of neoadjuvant chemotherapy for BRPC

**DOI:** 10.1097/JS9.0000000000002428

**Published:** 2025-04-29

**Authors:** Yuanzhi Fu, Junhao Chen, Shi Fu

**Affiliations:** a Department of Urology, The Second Affiliated Hospital of Kunming Medical University, Yunnan, China; b Kunming University of Science and Technology, Kunming, China

*Dear Editor,*

We read with great interest the article published in the *International Journal of Surgery*. He *et al*^[^1^]^ conducted a prospective study in which patient-derived organoids (PDOs) were cultured from tumor samples of BRPC patients, and drug sensitivity tests were performed using these PDOs to identify the most suitable chemotherapy drugs for each patient. Based on the results of the PDO-based drug sensitivity testing, the research team customized a neoadjuvant chemotherapy (NAC) regimen for patients, initially using the standard gemcitabine + nanoparticle albumin-bound paclitaxel (GnP) regimen, followed by adjustments based on PDO test results. The study showed promising resectability rates with PDO-based NAC in BRPC patients.

Pancreatic cancer is a highly malignant tumor originating in the pancreas, characterized by high mortality and low survival rates. According to the World Health Organization (WHO), the 5-year survival rate for pancreatic cancer is less than 6%, with most patients diagnosed at advanced stages where surgical resection is no longer possible. Borderline resectable pancreatic cancer (BRPC) refers to tumors that involve nearby major blood vessels (e.g., portal vein, superior vena cava, or arteries) but have not metastasized, allowing for a potential surgical resection. Without neoadjuvant therapy, the surgical resection rate for BRPC patients is typically low; however, after neoadjuvant therapy, the conversion rate from unresectable or borderline resectable to resectable is 31%. Although neoadjuvant therapy can shrink tumors and make them resectable, patients still face a high recurrence rate, particularly in the first 1 to 2 years post-surgery.

According to the NCCN and European Society for Medical Oncology (ESMO) guidelines, neoadjuvant chemotherapy (NAC) is a common treatment strategy for pancreatic cancer, especially for borderline resectable and locally advanced pancreatic cancer patients. The primary goal of this treatment is to shrink the tumor through chemotherapy to make it resectable. However, this approach has significant drawbacks, as approximately 40% of BRPC patients may still not achieve resectability after NAC, with the tumor continuing to progress locally or metastasize. Moreover, imaging assessments can be difficult, and prolonged treatment may delay surgery. The study relied on CT scans and RECIST criteria to assess the response to NAC but did not discuss potential inaccuracies in imaging evaluations. For example, after NAC, the tumor diameter was 2.2 cm, but it was not clarified whether fibrosis interference with imaging results was considered.

**HIGHLIGHTS**

- The imaging assessment may be inaccurate, as the interference of fibrosis on imaging results was not fully discussed, which could affect the actual tumor evaluation.
- Although adverse events (AEs) were reported, there was a lack of detailed management of these events, such as dose adjustments, treatment interruptions, or their impact on subsequent surgery.
- The study did not explore patient heterogeneity in depth, and individual differences may influence treatment response. Future studies should consider analyzing more patient subgroups based on characteristics like age and gender.
- While the primary endpoint (ORR) and secondary endpoints (R0 resection rate, etc.) were reported, the interpretation of results and statistical significance verification were insufficient. Multivariable analysis was not conducted, and potential confounding factors were not adequately controlled.

The study also reported that 42.1% (8/19) of patients experienced adverse events (AEs) of various degrees, including 26.3% with grade 2 myelosuppression and 5.3% with grade 3 myelosuppression, but the management of these AEs (e.g., dose adjustments, treatment interruptions) and their impact on subsequent surgery were not detailed. One patient died from a biliary infection, but the specific relationship between the infection and NAC toxicity was not analyzed. The lack of detailed toxicity management could limit clinicians’ confidence in applying this strategy in practice.

The patient population in the study may have some heterogeneity, as individual differences among BRPC patients could influence treatment responses. The study did not explore how these factors might affect the efficacy of neoadjuvant chemotherapy. Future studies should consider analyzing patient subgroups based on characteristics such as age and gender and perform stratified analyses to evaluate the impact of these factors on treatment responses. Additionally, while the study evaluated the primary endpoint (ORR) and secondary endpoints (R0 resection rate, postoperative complications, etc.) through statistical analysis, the interpretation of results and verification of statistical significance were insufficient. For instance, the data did not provide details on how potential confounding factors were controlled, nor was multivariable analysis conducted to assess the influence of different patient characteristics on treatment responses. Multivariable regression analysis helps control for confounders like age and comorbidities, providing a clearer assessment of treatment effects. For example, age is a critical factor influencing treatment outcomes and disease progression. Elderly patients often have lower drug resistance and may respond poorly to chemotherapy. Additionally, older patients are more likely to have other chronic conditions, such as hypertension and diabetes, which can affect their overall health status and tolerance to treatment. Failure to adjust for age may lead to an imbalance in treatment effects between different age groups, thereby affecting the accuracy and generalizability of the results.

I sincerely appreciate the authors’ outstanding and valuable contribution to advancing our understanding of the optimal treatment strategies for BRPC patients, and we highly recognize and praise the importance and value of their research findings.

## Data Availability

None.
